# Current and Potential New Targets in Systemic Sclerosis Therapy: a New Hope

**DOI:** 10.1007/s11926-020-00918-3

**Published:** 2020-06-19

**Authors:** Monique Hinchcliff, Steven O’Reilly

**Affiliations:** 1 Section of Rheumatology and allergy, Yale School of medicine, Yale University, New Haven, CT USA; 2 Department of Biosciences, Durham University, Stockton Road, Durham, UK

**Keywords:** Systemic sclerosis, Pathogenesis, Cytokines, Janus kinases, STAT3

## Abstract

**Purpose of Review:**

Systemic sclerosis (SSc) is an autoimmune connective tissue disease in which there is an activation of fibroblast to a myofibroblast that secretes huge amounts of extracellular matrix. Currently, no treatment exists that modifies the fibrosis elements and new therapeutic targets are badly needed. This review examines the current state of treatments and emerging therapeutics.

**Recent Findings:**

Nintedanib was found to significantly reduce the rate of decline in SSc associated FVC, although it has no benefit on skin fibrosis. New cannabinoid receptor2 agonist has shown superb effects in phase II and results in phase III are anticipated. Other targets are currently being tested in clinical trials and new targets that are yet to be tested are increasing in the SSc literature.

**Summary:**

Nintedanib is now licenced for SSc interstitial lung disease but this does not modify the skin fibrosis. Current ongoing trials will determine the role of various targets. New targets are emerging as we gain a deeper understanding of disease pathogenesis.

## Introduction

Systemic sclerosis (SSc) is an autoimmune idiopathic disease which is characterised by a specific triad of features; these are vasculopathy, inflammation and fibrosis [1–3] with a high case-specific mortality (Table 1). Fibrosis is a key component of the disease and is increasingly recognised as a key cause of morbidity and mortality in many diseases with organ-specific targets. Although tremendous strides have been made in understanding the biology of fibrosis, still no targeted therapies have been approved for fibrotic diseases and none especially in SSc.

**Table 1:** Clinical features of systemic sclerosis

Vasculopathy/Raynaud’s phenomenon	
Inflammation	
fibrosis	

Fibrosis is defined as the excessive deposition of fibrous tissue and extracellular matrix in an organ often in response to injury. This is chiefly mediated by a specific cell type termed the myofibroblast that becomes ‘activated’ in response to a multitude of factors that then endows the cell with resistance to apoptosis [4], increased contractility and exuberant expression of extracellular matrix (ECM) molecules including collagen and fibronectin [5]. This is conserved among whichever organ system is affected and is mainly the dermal and lung fibroblasts that are activated in SSc. The precise molecular mechanisms that govern activation of the myofibroblast are still not fully resolved but huge strides in our understanding have occurred in recent years [2, 6]. The aim of this review is to give an overview of current perspectives on pathogenesis and new possible therapeutic targets in a disease that currently has an unmet need.

## Fibrosis as a Concept

Accumulation of fibrosis tissue and ECM in an organ defines fibrosis. It is often in response to injury as a normal reparative response to restore homeostasis. The failure to terminate this wound healing response may underlie all fibrotic diseases. Damage to the tissue can come from a variety of diverse sources including infections, autoimmune reactions and physical damage. The normal wound healing response is normally initiated by damage to endothelial epithelial cells that induces the release of inflammatory mediators and begins clotting. This is followed by the release of platelet factors and chemokines that result in the recruitment of leukocytes that then release pro-resolving factors (such as IL-13) that facilitate repair and thus restore homeostasis [7]. Local fibroblasts are differentiated into myofibroblasts that express the marker α-smooth muscle actin and the increased deposition of ECM. This all results in the resolution of the wound, but if the rate of synthesis of ECM outweighs the rate of degradation, fibrosis ensues, which culminates in organ failure. It is suggested that around 45% of deaths in the Western world are attributed to a fibrosis component [8]. This means that fibrosis is currently a significant unmet need. SSc in particular has no therapies that target the fibrosis but recent discoveries are shedding light on the mechanisms that underlie the disease process.

## WNT Signalling as a Target in SSc

Wnt is a highly conserved signalling pathway that is involved in organ development [9]. Since Wnt was discovered over 35 years ago [10], there has been a major interest in this pathway in regard to development, cancer and most recently fibrosis [11, 12]. Enhanced Wnt signalling has been found in SSc with higher levels of the Wnt agonists both in the blood and tissues from patients [13–15].

Indeed, forced stabilisation of β-catenin, a main hub of Wnt signalling, in dermal fibroblasts, results in spontaneous fibrosis and increased collagen fibres in the mouse [14].

A recent clinical trial in SSc patients using C-82 to block Wnt signalling was well tolerated and showed reduction in a specific cluster of genes known to be associated with SSc; however, no clear clinical benefit was shown [16]. C-82 is an active metabolite of PRI-724, an inhibitor of Wnt that blocks catenin to its co-activator. Although C-82 had no clinical benefit, it could have been that the treatment regime needed longer to reverse long-standing fibrosis.

## The Endocannabinoid System

Cannabinoids are a diverse class of compounds that are structurally similar to the psychoactive compound derived from *Cannabis sativa*, THC. There are three classes of cannabinoids: endogenous cannabinoids produced endogenously in the body, phytocannabinoids that are plant-derived and synthetic cannabinoids that are generated in the laboratory [17].

This system employs two types of cannabinoid receptors CB1 and CB2. It has been shown that both CB1 and CB2 are upregulated in SSc dermal fibroblasts in culture and that incubation with the synthetic cannabinoid Win55,212–2 in vitro reduced collagen 1 production. This was also associated with a downregulation of TGF-β1 levels and the pro-fibrotic cytokine Interleukin-6 [18]. Win55,212–2 also abrogated dermal fibrosis in the bleomycin model of fibrosis [19] suggesting that this works in vivo. Indeed, stimulation of CB2 receptor with the agonist JWH133 reduced lung fibrosis in the bleomycin lung fibrosis model [20]. Ajulemic acid, a synthetic cannabinoid, is also anti-fibrotic in animal models of lung fibrosis [21]. It appears that the CB2 receptor is important for the therapeutic anti-fibrotic effects of cannabinoid agonists in SSc, and in keeping with this lenabasum, synthetic CB2R-specific agonists developed by Corbus Pharmaceuticals recently revealed in a phase II study that patients receiving lenabasum had a mean reduction of 9.2 points on the modified Rodnan skin score with no severe or serious adverse events recorded. The phase 3 trial is now underway with enrolment complete. These are eagerly anticipated as this appears a promising therapeutic.

## Serotonin/5HT

Serotonin is a neurotransmitter but is also released from activated platelets and exerts its effects via seven major receptor families. It has been demonstrated that in activated platelets from SSc patients, serotonin is released and binds to fibroblasts to induce tissue fibrosis and that this induction of tissue fibrosis is mediated through the 5-HT2b receptor [22]. This receptor is hugely overexpressed in SSc skin and blockade using the 5-HT2b-specific receptor blocker terguride and cyproheptadine-reduced fibrosis in animal models [22]. Interestingly, the serotonin receptor is also involved in activation of the hepatic stellate cell, the cell that is critical in liver fibrosis [23] and antagonism of the 5-HT2b receptor–reduced liver fibrosis in experimental liver fibrosis models [24]. Terguride is already licenced for use. In a phase II proof of concept study which included 12 patients that received terguride, there was a significant improvement in mRSS and a significant reduction in a 4 gene biomarker signature (ACR abstract 2016). A phase III trial is now underway.

## Targeting of Nuclear Receptors

Nuclear receptors are a family of transcription factors that appear critical in fibrosis [25]. Among these, peroxisome proliferator–activated receptors (PPAR) are critical in SSc [26]. PPAR-γ is a key nuclear receptor that is activated by fatty acids that are downregulated in SSc cells and tissues and PPAR-γ agonist rosiglitazone reduces fibrosis in animal models of SSc [27, 28]. Suggesting that rosiglitazone could be useful in SSc. Rosiglitazone is already licenced. Recently, a pan PPAR agonist molecule has been developed and was found to be potently anti-fibrotic in multiple animal models of fibrosis [29]. This pan agonist was even effective in lung fibrosis and pulmonary hypertension in the Fra2 mouse model [30]. A phase IIb clinical trial of IVA 337/lanifibranor in diffuse SSc patients failed to show a significant improvement in skin scores compared with that in the placebo (NCT02503644), which may have been attributed to background immunosuppressant therapy. Another nuclear receptor was also found to be critically important in systemic sclerosis and organ fibrosis. This receptor is called NR4A1 and is an orphan nuclear receptor as no ligand has to date been identified and the deletion of this gene in mice leads to leukaemia [31]. It was found in SSc that NR4A1 is a negative regulator of fibrosis and that temporary upregulation of this receptor via TGF-β1 leads to a downregulation of fibrotic genes that this is dysfunctional in SSc and this ultimately leads to skin fibrosis [32]. In animal models of disease, it was shown that this lack of responsiveness to negative feedback can be overcome with NR4A1 agonists [32]; thus, these agonists of NR4A1 could be a possible therapeutic target in SSc.

## Targeting of Interleukin-6

IL-6 is a classic pro-inflammatory cytokine that is also potently pro-fibrotic [33]. The levels of IL-6 are higher in SSc blood and also in the tissues. IL-6 is pro-fibrotic through what is termed trans-signalling, which is through binding of the soluble form of the IL-6 receptor (fibroblast do not express this) and IL-6 with gp130 to initial signalling [34]. We have shown that the increase in collagen and other ECM is due to downstream STAT3 signalling [35, 36], suggesting that IL-6 is a therapeutic target in the disease. Based on these in vitro findings, animal models [37] and a few case reports [38] using an antibody that binds the IL-6 receptor, tocilizumab, a clinical trial was undertaken. The primary endpoint was not met in that there was not a significant improvement in the change from baseline to week 48 modified Rodnan skin score (mRss); however, there were clinically meaningful changes in lung function [39]. Therefore, although the skin score did not change significantly compared with that in the placebo, the lung function at least stabilised with less decline in FVC [39, 40]. If the endpoint was different, this would likely be licenced. It is unlikely that this will now be licenced for SSc. This was a well-designed clinical trial that was enriched for early diffuse disease with high CRP and lessons have been learned. One study used skin explants from this trial and RNA sequencing to identify differentially expressed genes in an unbiased way and this amazingly showed that tocilizumab treatment compared with the placebo resulted in a huge reduction in TGF-β-target genes [41], showing that it did indeed target pro-fibrotic pathways, but maybe these are more responsive in the lung [33, 41].

## Interleukin-13

IL-13 is a cytokine that was found to promote IgE class switching and inhibit pro-inflammatory cytokines. It is now recognised to play a major role in allergy and fibrosis and particularly SSc [42]. We and others have found that T cells in SSc produce high levels of IL-13 and this T cell–derived IL-13 is directly pro-fibrotic [43, 44]. SAR156597 is a monoclonal antibody targeting IL-4 and IL-13 and a phase II proof of concept placebo-controlled trial is now ongoing (ClinicalTrials.gov; NCT02921971) in SSc and a phase II trial has been undertaken in idiopathic pulmonary fibrosis, which did not have positive results [45].

## Interleukin-31

IL-31 was first described in 2004 as a four alpha-helix IL-6 family cytokine that is synthesised by activated Th2 cells [46]. IL-31 signals through IL-31 receptor A and oncostatin M receptor. IL-31 is associated with pruritus, a common symptom in SSc and signals through a STAT3 mechanism, similar to IL-6. We have found high levels of IL-31 in SSc serum compared with that in healthy controls and that this is directly pro-fibrotic (in press, Rheumatology 2020). We also show that this is mediated through phosphorylation of STAT3 and its receptor is regulated epigenetically in SSc. Furthermore, mini-pump delivered IL-31 in mice led to substantial skin fibrosis. Nemolizumab is a monoclonal antibody that targets the IL-31 receptor and has been shown to be helpful and relieve the itch in atopic dermatitis [47].

## Nintedanib

**Fig. 1:**
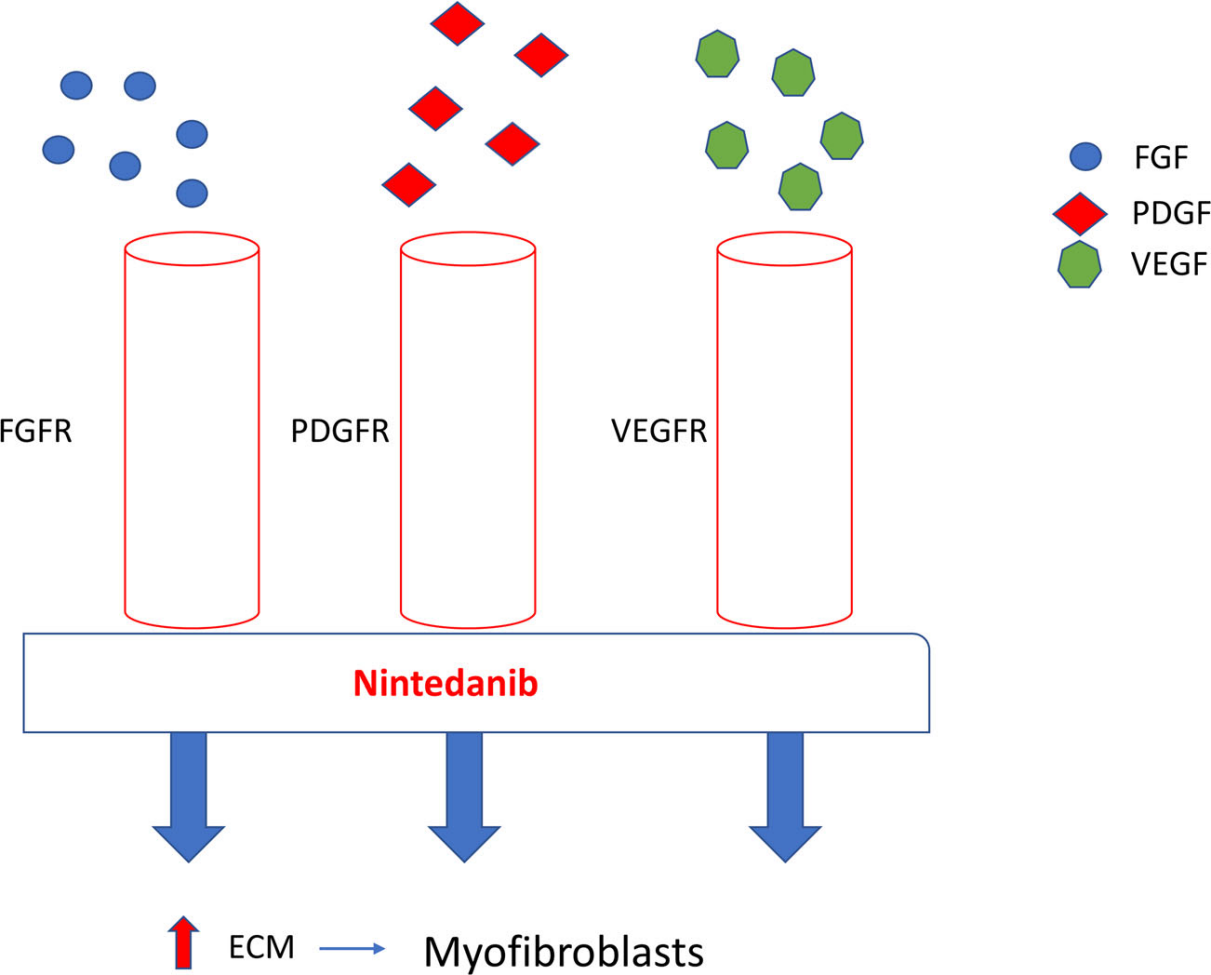
Mechanism of action of nintedanib in lung fibrosis in SSc. Nintedanib work by binding to the ATP binding pocket FGF, PDGF and VEGF receptors resulting in blockade of the autophosphorylation of these receptors and subsequent downstream signalling pathways. This approach blocks pro-fibrotic and proliferative pathways downstream of the receptors stimulation which includes Src and Ras (not shown). This multiplicity of the targets may underpin is therapeutic efficacy. FGF fibroblast growth factor, PDGF platelet-derived growth factor, VEGF vascular endothelial growth factor

Nintedanib is a small molecule tyrosine kinase inhibitor acting on the PDGF, VGF and VEGF receptors and thus targets cytokine-induced activation of fibroblasts (Fig. 1). Nintedanib has been shown to reduce fibrosis in animal models of SSc [48]. Nintedanib recently became the first to be FDA-approved for SSc interstitial lung disease for slowing the rate of decline in pulmonary function [49]. The SENSCIS trial is a phase III, randomised, placebo-controlled trial of nintedanib vs placebo in which 576 patients were recruited. The results demonstrated that those who received nintedanib had a lower rate of change of FVC compared with the placebo (− 52.4 ml per year vs − 93.3 ml per year; difference 41 ml *P* = 0.04), with no change in the mRss [49]. This is the first approved treatment and signifies an important milestone at least in treating lung diseases decline.

## Abatacept

It is clear that the immune system is involved in SSc and in particular, T cells play a prominent role in early disease [3, 43]. Abatacept is a fusion protein of the Fc region of IgG1 and the extracellular domain of cytotoxic T lymphocyte–associated antigen 4 (CTLA-4). This blocks the interaction of CD28 on the T cell and CD80/86 on the dendritic cell and is approved for RA and appears to have a good safety profile [50]. Based on this, a phase II trial was undertaken in early diffuse SSc patients; this showed that abatacept was well tolerated but that the change in mRss was not significant [51]; however, there was a greater decline in mRss in the abatacept group.

## Targeting B Cells

Given that SSc is associated with specific auto-antibodies, this suggests that B cells are intimately involved. Rituximab is a monoclonal antibody to CD20 and its mechanism of action is thought to be depletion of B cells [52]. A study from the EUSTAR database suggested that there was a reduction in skin score and also prevention of worsening of FVC [53]. In a larger observational study, patients with SSc treated with rituximab had a reduction in skin fibrosis but no significant prevention of worsening of lung function [54]. Further studies are warranted into the effects of rituximab, particularly if there is any benefit in using with background MMF.

## Interferon

Anifrolumab is an antibody against the type I interferon receptor. In a phase I multicentre, open-label study in SSc patients, it was found to be safe and tolerable but not efficacy was assessed [55].

## Bardaloxone Methyl

Bardaloxone methyl is currently being tested in a clinical trial for SSc associated pulmonary hypertension (ClinicalTrials.gov; NTC02657365). Bardaloxone methyl is a nrf-2 stimulator, which should reduce oxidative stress and fibrosis. Nrf-2 is a critical stress response gene that is reduced in SSc fibroblasts and nrf-KO mice have enhanced fibrosis [56], indicating it plays a key role in fibrosis.

## Abituzumab

Abituzumab is a humanised IgG2 antibody targeting γν subunit (CD50) integrins. Integrins are critical transmembrane receptors that facilitate ECM interactions and signal transduction and can activate TGF-β. a key pro-fibrotic molecule. Importantly, integrin ανβ5 is upregulated on SSc dermal fibroblasts and upregulation of this integrin leads to enhanced deposition of collagen [57, 58]. A phase II trial of abituzumab is currently recruiting about 180 patients with SSc and interstitial lung disease with and FVC 40–85% predicted (ClinicalTrials.gov; NCT02745145). The primary endpoint is the change in baseline in absolute FVC at week 52.

## Janus Kinase Inhibition

Janus kinases (JAKs) are non-receptor tyrosine kinases and there are 4 JAKs in mammals: Jak1, Jak2, Jak3 and Tyk2. They transduce cytokine signals via phosphorylation of STATs. Tofacitinib is a JAK1 and JAK3 inhibitor and is licenced for rheumatoid arthritis [59]. In SSc, we have found that blockade of JAK signalling in vitro reduced cytokine-mediated increased collagen expression [35] due to the fact it blocked downstream STAT3 signalling, a key event in fibrosis. Selective JAK2 inhibitors also reduced collagen in SSc fibroblasts and in vivo in multiple animal models and peripheral blood mononuclear cells have constitutively active JAK levels and peficitinib, a JAK inhibitor, reduced lymphocyte activation in these cells [60]. Tofacitinib is currently undergoing clinical trials in diffuse SSc in a phase I/II randomised trial. In a recent abstract, there was a trend towards improvement in the mRss (Khanna et al. ACR 2019). Table 2 lists the currently available JAK inhibitors approved for inflammatory disease.

**Table 2:** Approved JAK inhibitors for autoimmune disease

Name	Specificity
Tofacitinib	Jak1/3> Jak2, Tyk2
Ruxolitinib	Jak1/Jak2> Tyk2
Baricitinib	Jak1/Jak2
Upadacitinib	Jak1

## Different Strokes for Different Folks

In the last few years, there has been an explosion of information on the understanding of molecular pathways at work in SSc. This has led to the development of new therapeutics, but to date, all of the clinical trials have failed their primary endpoint except for the SENSCIS trial. This may be due to the fact that whilst all the patients may have SSc ‘per se’, they may have different molecular drivers. In other words in say, for instance, the tocilizumab trial, many may have had a high IL-6 STAT3 signature and thus responded, but some may have had a disease that was driven by JAK3 instead or IL-13. Determining target activation prior to clinical trial may tailor the treatment more effectively to the target. Quite how you do this may be difficult. In terms of an ‘IL-6 driven disease’, do you take serum IL-6 as the biomarker or tissue phosphorylated STAT3 which is likely to be a better marker of disease that blood levels? At what time do you measure the analyte? Given circadian oscillations in inflammation, timing of sample collection may be critical. What if multiple pathways are at work at one time? It could be that combination therapy is important.

## Conclusion

SSc is a complex heterogenous disease whose clinical course is unpredictable. Recently, nintedanib has been approved for a lung disease associated with SSc, but this does not modify the skin fibrosis. Many new targets are emerging, and this review has highlighted some of these. It may well be that target identification of the molecular pathway prior to entering the clinical trial for a designated target helps enrich.
